# Drosophila Polypyrimidine Tract-Binding Protein (DmPTB) Regulates Dorso-Ventral Patterning Genes in Embryos

**DOI:** 10.1371/journal.pone.0098585

**Published:** 2014-07-11

**Authors:** Joseph Heimiller, Vinod Sridharan, Jim Huntley, Cedric S. Wesley, Ravinder Singh

**Affiliations:** 1 Department of Molecular, Cellular and Developmental Biology, University of Colorado, Boulder, Colorado, United States of America; 2 BioFrontiers Next-Gen Sequencing Facility, University of Colorado, Boulder, Colorado, United States of America; 3 Departments of Genetics and Medical Genetics, University of Wisconsin, Madison, Wisconsin, United States of America; Roswell Park Cancer Institute, United States of America

## Abstract

The Drosophila polypyrimidine tract-binding protein (dmPTB or *hephaestus*) plays an important role during embryogenesis. A loss of function mutation, *heph^03429^*, results in varied defects in embryonic developmental processes, leading to embryonic lethality. However, the suite of molecular functions that are disrupted in the mutant remains unknown. We have used an unbiased high throughput sequencing approach to identify transcripts that are misregulated in this mutant. Misregulated transcripts show evidence of significantly altered patterns of splicing (exon skipping, 5′ and 3′ splice site switching), alternative 5′ ends, and mRNA level changes (up and down regulation). These findings are independently supported by reverse-transcription-polymerase chain reaction (RT-PCR) analysis and *in situ* hybridization. We show that a group of genes, such as *Zerknüllt*, *z600* and *screw* are among the most upregulated in the mutant and have been functionally linked to dorso-ventral patterning and/or dorsal closure processes. Thus, loss of dmPTB function results in specific misregulated transcripts, including those that provide the missing link between the loss of dmPTB function and observed developmental defects in embryogenesis. This study provides the first comprehensive repertoire of genes affected *in vivo* in the *heph* mutant in Drosophila and offers insight into the role of dmPTB during embryonic development.

## Introduction

RNA-binding proteins regulate many aspects of post-transcriptional gene expression. The heterogeneous nuclear ribonucleoproteins (hnRNPs) are ubiquitously expressed, associate with nascent transcripts, and play various roles in basic RNA metabolism [1], [2]. One such protein, the polypyrimidine-tract-binding protein (PTB or hnRNP I), binds to pyrimidine-rich sequences containing motifs such as UCUU and UUCU [3]–[7]. It affects mRNA splicing, polyadenylation, translation, mRNA stability/degradation, and mRNA localization (reviewed in [2], [8]–[10]). In humans, of the three PTB genes (PTBP1, PTBP2, and PTBP3), PTBP1 has near-ubiquitious expression, whereas PTBP2 and PTBP3 have tissue-specific expression [11].

*Drosophila melanogaster* has only one PTB ortholog, *hephaestus* (*heph*). Different Drosophila *heph* mutants have wide-ranging phenotypes, including embryonic lethality, sensory bristle and wing margin abnormalities, and sterility in adult males [6], [12]–[14]. Genetic screens and analysis have implicated the *heph* gene or dmPTB protein in *oskar* mRNA translational repression during oogenesis [15], in efficient Grk signaling in the germline [16], and in Notch signaling, for example, to repress *Notch* activity or pathway following ligand-dependent activation during wing development and embryogenesis [12], [17]–[22]. Very little is known, however, about the role or the downstream targets of dmPTB in these various developmental processes in Drosophila.

We took an unbiased genome-wide approach to study the role of dmPTB in Drosophila embryos through transcriptome profiling. We used RNA-Seq analysis of embryonic mRNAs and show that loss of *heph^03429^* function results in misregulation of numerous transcripts, including those known to be functionally involved in dorso-ventral patterning and dorsal closure during embryogenesis.

## Results

### High throughput sequence analysis for transcriptome profiling

As noted above, the molecular basis of the developmental defects in the *heph^03429^* mutant remains unknown. The *heph^03429^* is a null allele or an extreme hypomorph (below detectable level) [12], [15]. We therefore used high throughput sequencing as an unbiased approach to identify misregulated mRNAs in the mutant. We performed the RNA-Seq protocol on polyadenylated RNA from the *heph* ^03429^ mutant and wild-type control (*yw*) embryos. We obtained 175,257,719 sequence reads (100-nucleotide length) for the wild-type control and 167,968,932 sequence reads for the *heph* ^03429^ mutant. Using TopHat, over 90% of the sequences could be mapped to the Drosophila genome and about 14% corresponded to splice junction reads (Table 1). Multiple sequence reads from the *heph* ^03429^ mutant confirmed the insertion of the transposon *P* element in the *heph* locus (Figure S1). About 90% of genes were represented. We observed that, of the total 14,794 genes, about 1520 genes showed no sequence reads (1359 for the mutant), about 2652 genes (2799 for the mutant) had <1 FPKM (Fragments per kilobase per million) reads, about 1720 genes (1796 for the mutant) had between 1 and 5 FPKM, about 4414 genes (4202 for the mutant) had between 5 and 25 FPKM and about 4488 genes (4638 for the mutant) had more than 25 FPKM. We conclude that the majority of genes are represented by the sequence reads.

**Table 1:** Sequencing and mapping statistics for the *heph^03429^* analysis.

Group	Number of reads	Number of spliced reads	Reads mapped to genome (%)
*yw* embryos (control) replicate 1	28,158,603	2,331,740 (8%)	91%
*heph^03429^* embryos replicate 1	27,820,399	2,395,023 (9%)	92%
yw embryos (control) replicate 2	175,257,719	23,774,538 (14%)	91%
*heph^03429^* embryos replicate 2	167,968,932	23,383,095 (14%)	92%

### Transcriptome profiling identifies misregulated isoforms

**Figure 1:**
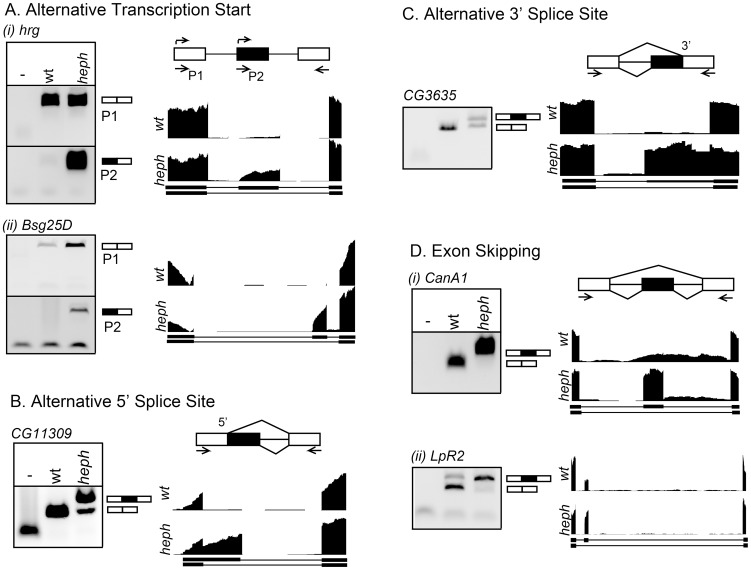
mRNA isoforms misregulated in the *heph* ^03429^ mutant. RT-PCR analysis for alternative isoform usage between the wild-type control and the *heph* ^03429^ mutant. Schematics are shown for each alternative isoform category. **A.** Alternative transcription start sites, **B.** Alternative 5′ splice site, **C.** Alternative 3′ splice site, and **D.** Exon skipping. Primers used for RT-PCR are indicated. RNA-Seq read pileups across the alternatively expressed section of each gene are shown for the wild-type (yw) control and the *heph^03429^* mutant.

We analyzed the *wild-type* control and *heph^03429^* transcriptomes for mRNA isoform and expression level differences. With respect to mRNA isoforms, differences could arise from transcription start sites, alternative 5′ splice sites, alternative 3′ splice sites, and/or exon skipping. We manually inspected over 100 candidates from sequence analysis algorithms and selected six of them based on fold difference and isoform type for analysis by RT-PCR. We observed significant isoform differences for each category: Use of alternative 5′ regions from transcription start sites was observed for *hrg* and *Bsg25D* genes such that the proximal transcription start site was used only in the *heph* ^03429^ mutant (Fig. 1A, i and ii). Alternative 5′ splicing resulted in use of a downstream 5′ splice site and thus a longer transcript for *CG11309* in the *heph* ^03429^ mutant (Fig. 1B). Switching to an upstream 3′ splice site resulted in a longer transcript for *CG3635* in the *heph* ^03429^ mutant (Fig. 1C). For the *CanA1* transcript, an exon that was skipped in the wild-type control was included in the *heph* ^03429^ mutant (Fig. 1D, i). Finally, there was a quantitative switch in the ratio of *LpR2* transcripts from the predominantly exon-skipped isoform in the wild-type control to the predominantly exon-included isoform in the *heph* ^03429^ mutant (Fig. 1D, ii). Based on our analysis, these are the most significant isoform differences in the *heph* ^03429^ mutant. It is possible that further scrutiny may reveal a few more examples of small quantitative differences in alternative transcripts in the *heph* ^03429^ mutant. Differences in poly(A) site usage were not analyzed (see the Materials and Methods section). These findings show that the loss of dmPTB function in the *heph* ^03429^ mutant resulted in significant differences in mRNA isoforms for specific transcripts.

### Transcriptome profiling identifies expression level differences

**Figure 2:**
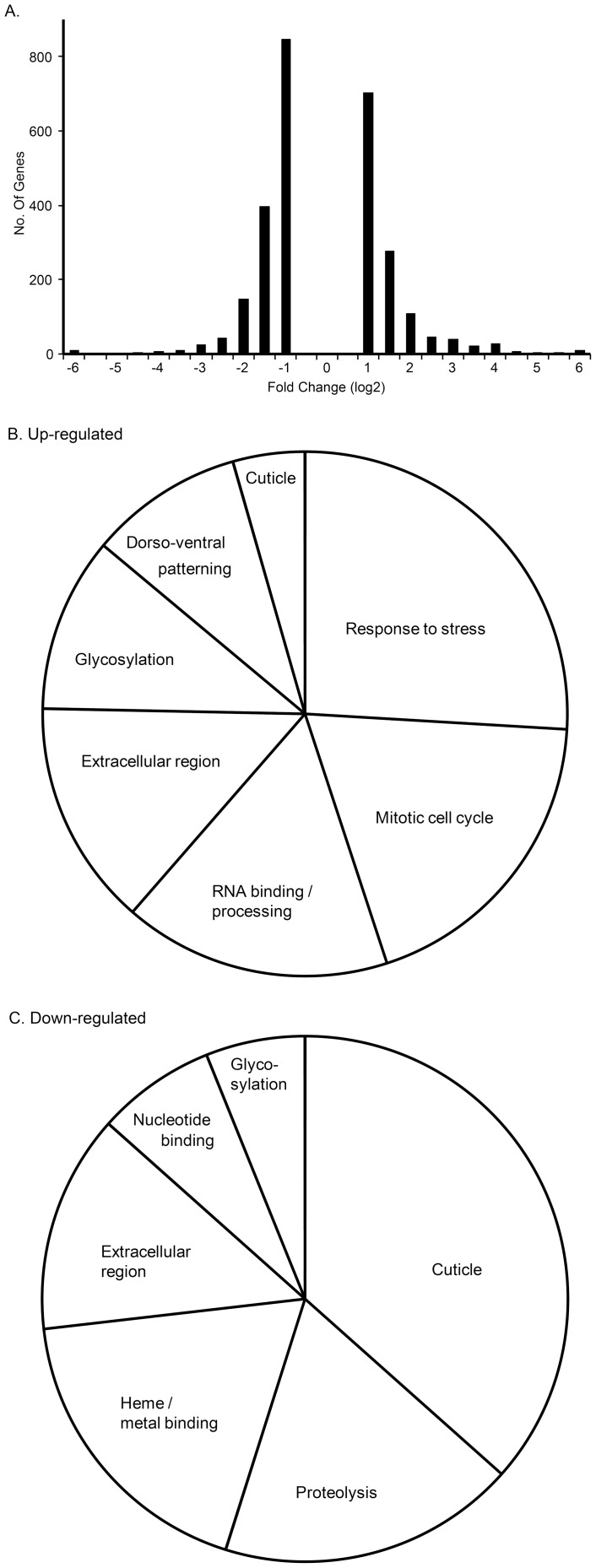
Analysis of RNA-Seq differential expression between wild-type control and *heph^03429^* mutant. A. Histogram depicting fold-change values (above two-fold) of all genes that are significantly changed. **B and C.** Most overrepresented (four-fold and above up-regulated or down-regulated) gene ontology categories from the DAVID analysis are shown by their percent representation in terms of number of genes. Many genes belong to multiple categories.

We compared expression levels for each gene between the two samples (Fig. 2A). Of the total 14,794 genes, 1161 genes showed no expression in either sample. 6419 genes were called significant by the algorithm, however, 3709 were below the two-fold cutoff. The remaining 2710 genes contained four snRNA/snoRNA gene categories and five genes had a value of zero (as denominator to preclude fold-change calculation), which were removed from further analysis. Approximately 2217 genes had a difference of 2-3 fold (Fig. 2A). There was over four-fold upregulation for 255 genes and downregulation for 229 genes. Six genes changed by 128-fold or more. These results show significant changes in gene expression of specific genes in the *heph* ^03429^ mutant.

Next, we asked if some functional gene ontology categories were over-represented in the differentially expressed genes. We subjected the 463 genes that showed over four-fold difference in expression levels between the wild-type control and the *heph* ^03429^ mutant to gene ontology analysis using DAVID; 20 genes were not recognized and thus ignored. We selected the top categories from the output of approximately 240 genes, including 158 in the up-regulated and 82 in the down-regulated set. Our analysis included genes relevant for RNA-binding, cell cycle, stress, signaling, structural components, and axis formation or dorso-ventral patterning (Fig. 2B). Genes relevant for cuticle synthesis were among the most down-regulated (Fig. 2C). These results are consistent with the idea that dmPTB is a potent upstream regulator because it affects numerous cellular functions.

### Genes related to dorso-ventral patterning, amnioserosa development or dorsal closure, and cuticle formation are among the most misregulated

We next analyzed components of specific functional categories. Consistent with the previous observations that *heph* regulates Notch signaling, we found that there was a 75% increase in the *Notch* transcript level in the *heph* ^03429^ mutant. Components of the Notch signaling such as *rumi*, *enhancer of split m4* and *Brother of Bearded* also showed an increase (Table 2). Genes in other specific functional categories were also severely misregulated in the mutant. For example, several genes that had been functionally linked to dorsal/ventral embryonic axis formation and amnioserosa development such as *screw*, *twisted gastrulation* and *zerknüllt* (*zen*) showed over 16-fold upregulation (Table 2). Many others such as *Protein Z600* and *spindle E* have also been functionally linked to dorso-ventral patterning and showed a 19-fold and 9-fold increase, respectively [23], [24]. In addition to these up-regulated transcripts, we identified many genes that were considerably down-regulated (up to 165-fold). Many of these genes relate to cuticle development, dorsal closure, proteolysis, signal peptide and the extracellular region (Table 2). We conclude that in addition to the smaller differences observed for *Notch* signaling-related transcripts, many more transcripts, with much larger differences, are involved in dorso-ventral patterning, amnioserosa development and cuticle formation.

**Table 2:** List of selected, differentially expressed genes in the *heph^03429^* mutant, relevant for dorso-ventral axis specification, Notch signaling and cuticle formation.

Gene name	Fold change	Ontology
screw	27	Axis, AS
shrew/CG11582	21	DC, AS
zerknüllt	20	D/V, AS, CU
Protein Z600	19	D/V
twisted gastrulation	16	Axis, AS
spindle E	9	D/V
nullo	9	morph.
Cuticular protein 47Eb	9	CU
gastrulation-defective	8	D/V
Brother of Bearded A	6	Notch
krimper	6	D/V
squash	5	D.C.
Spook	5	D.C., CU
rumi	5	Notch
Oskar	5	Axis
zerknüllt-related	5	D/V, AS
swallow	5	Axis
Shade	4	D.C., CU
mummy	3	D.C.
e(spl) m4 (Bearded family)	2	Notch
Cuticular protein 5C	−5	CU
Cuticular protein 78Cc	−5	CU
Cuticular protein 65Ec	−6	CU
Cuticular protein 49Ae	−10	CU
Adult cuticle protein 1	−16	CU
Cuticular protein 49Ad	−165	CU

Genes are sorted by fold-change. Genes that are related to dorsal/ventral axis formation with less than four-fold change are not shown: *tolloid, jumu, wntD, Cg25C, mmy, rl, cactin, dib, Ptp61F, Dif, Src64B, aop, vrt, ImpE1, Abl, zip, Btk29A, ena*, and *chic*. Similarly, genes that are related to Notch signaling with smaller than four-fold change are not shown: *O-fut1, bib, sens, Sca, Su(H), CG13465, Ocho, Ebi, nct, Rpd3, Tom, m4, Dl, CG8027, Bre, neur, phyl, Al, elB, malpha, gro, amx, N, Rtf1, Brd, CtBP, dsh, Hs3st-B, HLHgamma, bun, sim*, and *e(spl)*. CU  =  cuticle, D.C.  =  Dorsal Closure, axis  =  axis specification, D/V  =  dorsal/ventral axis specification, morph.  =  embryonic morphogenesis, AS  =  amnioserosa.

### RT-PCR analysis confirms misregulated transcripts

**Figure 3:**
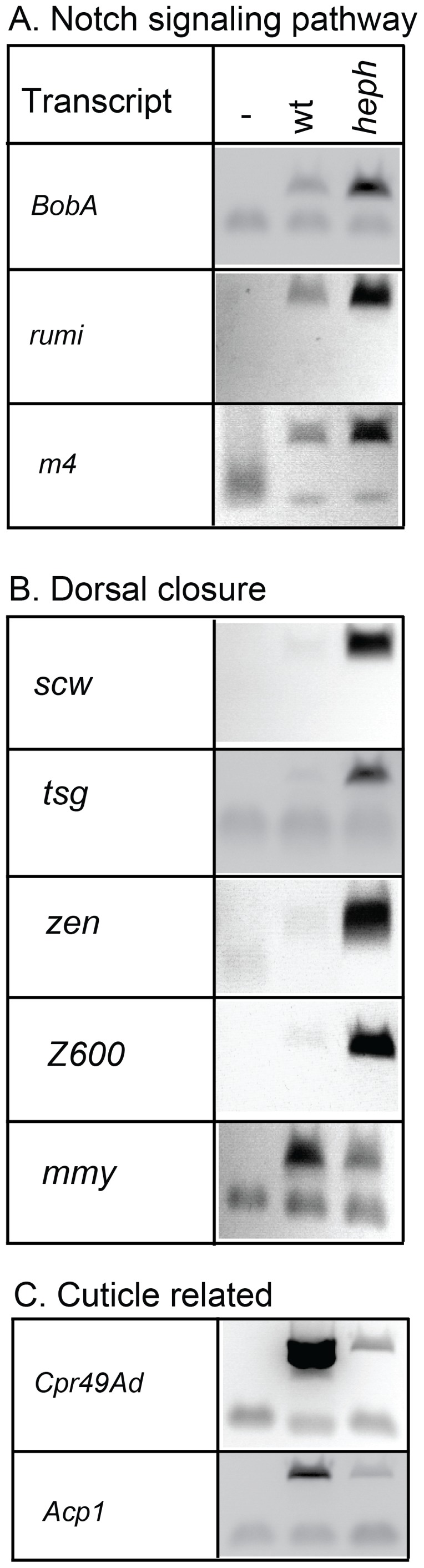
Analysis of differentially expressed genes in the heph^03429^ mutant using RT-PCR. Candidates relating to Notch signaling, dorso-ventral axis specification and cuticle formation were randomly picked for analysis. Three of the primer pairs tested did not work.

We randomly selected several candidates from specific gene ontology categories for analysis by RT-PCR for their expression level differences between the wild-type and mutant embryos. Fig. 3 shows that three components of the Notch signaling (*BobA*, *rumi*, and *m4*) and four candidates (*scw*, *tsg*, *zen*, and *Z600*) relevant to embryonic development (dorsal closure, axis formation, and/or dorso-ventral patterning) all showed upregulation, consistent with the differences observed from high throughput sequencing. Finally, two of the candidates related to cuticle structure or formation (*Cpr49Ad* and *Acp1*) recapitulated downregulation observed from the Illumina sequencing reads. We conclude that RT-PCR and high throughput sequencing independently confirm misregulation of specific transcripts in the *heph* ^03429^ mutant.

### Misregulation of zen and its link to dorso-ventral patterning defects

**Figure 4:**
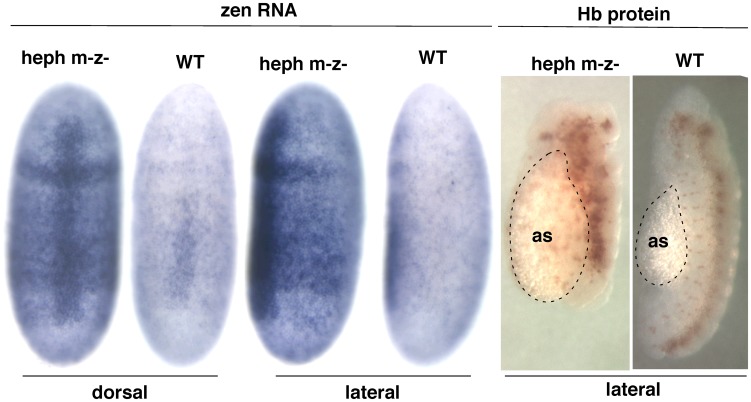
Expression of the *zen* mRNA is up-regulated in the dorsal and lateral regions of embryos in the *heph^03429^* mutant versus wild-type (WT). Right panel: Hyperplasia of the dorsal tissue, amnioserosa, in the mutant, which is expected from increased expression of dorsal fate genes such as *zen*.

Several components of the dorso-ventral patterning, axis formation, or proper development of amnioserosa are among the highest up-regulated genes. Proper embryonic developmental process involves establishment of Toll and Dpp signaling gradient slopes in opposite directions along the dorso-ventral axis, leading to cell fate specification along this axis [25], [26]. In this context, both temporal and spatial expression patterns are the key to proper development. This important feature of the spatio-temporal expression pattern is lost during RNA isolation from homogenized embryos. Therefore, to investigate the misregulation of *zen*, which is functionally linked to dorso-ventral patterning [27], we analyzed the expression pattern of *zen* in the wild-type control and the *heph* ^03429^ mutant embryos using *in situ* hybridization. We found that the *heph* ^03429^ mutant (maternal and zygotic null) showed significantly higher expression of *zen* as compared to the control embryos (Fig. 4, left panel), which is consistent with the RT-PCR result. This is true for both dorsal and lateral views. We also analyzed these embryos for hyperplasia of the dorsal tissue (amnioserosa), which is expected as a consequence of overexpression of *zen* [28]. Indeed, there was evidence of hyperplasia of the dorsal tissue (Fig. 4, right panel). We conclude that the *heph* ^03429^ mutant shows increased *zen* expression, reflected in the hyperplasia of the dorsal tissue, and developmental defects during dorso-ventral axis formation/patterning.

## Discussion

Our high throughput sequencing provides the first unbiased view of the altered transcriptome in the *heph^03429^* mutant embryos. We identify specific transcripts that are misregulated in the mutant, resulting from differences in either mRNA isoforms or mRNA levels. Transcript isoforms arise from differences at their 5′ ends, alternative 5′ splice site use, alternative 3′ splice site use, or exon skipping. These differences result in altered coding or non-coding regions of mRNAs. Also, we identify transcripts that show either significant up-regulation or down-regulation. Below we describe how these findings have contributed to our understanding of dmPTB/heph regulation and to cellular and developmental processes that are affected in the mutant.

### dmPTB as a regulator of canonical and non-canonical Notch signaling

dmPTB was previously shown to function as a suppressor of *Notch* signaling in the context of wing development and embryogenesis. Our sequencing confirms that Notch mRNA is up-regulated (75% increase) in the mutant. We argue that this increase is functionally consequential because *Notch* expression is tightly regulated as a change in *Notch* gene dosage significantly perturbs cellular and developmental processes [29]. We show that the effect of the dmPTB regulation on *Notch* mRNA levels is further amplified by the known downstream components of the canonical Notch signaling pathway (*rumi*, *enhancer of split m4* and *BobA*). This pathway would be most relevant to regulation in the neurogenesis in the ventral region of embryos and in cuticle development.

The *heph^03429^* embryos manifest high levels of filamentous or F-actin protein expression in the lateral region [22]. Therefore, we asked if the actin transcript is affected. We found very small difference (<2 difference) in the expression of actin genes (Actin79B, Actin88F, and Actin42A). Since actin mRNA levels cannot explain the defects in actin processes during embryogenesis, we explored if any regulator of actin dynamics is affected. Indeed, *bottleneck* (26-fold), *nullo* (10-fold), and *Sry-alpha* (8-fold) are significantly over-expressed in the *heph^03429^* mutant. These differences suggest that regulation of actin translation, stability, and/or polymerization, rather than actin mRNA levels, underlies the F-actin over-expression phenotype in *heph^03429^* embryos.

We are particularly drawn to several other genes that are dramatically upregulated (over 20-fold): *scw*, *tsg*, *zen*, *Z600 genes* belong to cellular and developmental processes linked to dorso-ventral patterning, dorsal closure, and/or amnioserosa development. The *zen* transcripts show spatio-temporal expression pattern reminiscent of dorso-ventral patterning disruption and amnioserosa hyperplasia. It is overexpressed in the dorsal region of *heph* embryos (Fig. 4).

How does dmPTB regulate *zen*? It is possible that the regulation of *zen* and other responsive genes is mediated through Notch signaling. Both canonical Notch signaling in the nucleus and the non-canonical Notch signaling are up-regulated in *heph^03429^* embryos [22], [30]. The canonical Notch signaling involves ligand-dependent processing, release, and translocation into the nucleus of the Notch intracellular domain (N^intra^/NICD) to activate target genes [31]–[33]. Up-regulation of canonical Notch signaling is associated with the development of the anti-neurogenic phenotype [34]–[36]. The *heph* embryos manifest the anti-neurogenic phenotype due to increased canonical Notch signaling [22]. We believe that this anti-neurogenic phenotype is an unlikely explanation for how dmPTB could affect *zen* regulation because this signaling suppresses development of dorsal tissues such as the amnioserosa [22]. We propose that the observed increase in *zen* mRNA possibly involves up-regulation of a non-canonical Notch pathway that promotes the expression of the phosphorylated form of Cactus, the negative regulator of Dorsal activity. This non-canonical Notch signaling activity involves Protein Kinase C function and promotes the specification of dorsal cell fates and the lateral epidermis [30]. Since Dorsal is a known repressor of *zen* [28], it is possible that *zen* upregulation in the *heph^03429^* embryos could be a consequence of Cactus-mediated suppression of Dorsal function. The involvement of Cactus up-regulation in altering mRNA expression in *heph^03429^* embryos is further supported by a 50% reduction in the expression of Dorsal-like immunity factor (Dif) [37]. The non-canonical Notch pathway could explain how the *heph^03429^* mutation results in *zen* upregulation and hyperplasia of the amnioserosa, affecting the lateral regions of the embryo.

### dmPTB affects many processes during embryogenesis

Intriguingly, genes relevant to cuticle formation were found to be the most down-regulated genes in the *heph^03429^* mutant. It is possible that the effect on cuticle genes/components is a reflection of excess canonical and non-canonical Notch signaling that disrupts the development of the larval cuticle during embryogenesis. Unfortunately, the phenotype of the *heph* ***^−^*** *N* ***^−^*** zygotic double mutant is a complex mosaic of neurogenic and anti-neurogenic phenotypes [22]. Therefore, it remains possible that some genes misregulated in the *heph^03429^* mutants are independent of the canonical and non-canonical Notch signaling mechanisms. Since there are changes in many components of the Wingless/Wnt pathway, for example WntD (Wnt inhibitor of Dorsal), it is possible that *heph* affects Wnt functions in addition to Notch functions. The Wingless/Wnt pathway is also involved in many aspects of the patterning of the developing larval epidermis [38]. The specific candidate genes identified in our analysis provide an important molecular handle to distinguish between these possibilities using appropriate genetic backgrounds. For example, analyzing the candidate genes in *Notch* and *wingless* mutant backgrounds might identify dmPTB-regulated genes specific to the Notch signaling pathways or the Wingless signaling pathway.

Our expression profiling between the wild-type and the mutant embryos clearly shows that there are additional gene ontology categories that are affected, including genes relevant to stress, gene regulation, and cell cycle. While the biological significance of all of these differences remain unclear, the effect on specific gene ontology categories likely holds clues to the role of dmPTB in various aspects (cell-type, tissue-specific, temporal, and spatial) of embryonic development (Fig. 2B).

### Regulatory mechanisms

The differences in mRNA levels, discussed above, could result from the effect on transcription/synthesis, mRNA degradation, or both. We note that some of the alternative transcripts could also be subject to degradation by the nonsense-mediated decay machinery. Although the effect of an RNA-binding protein directly on alternative transcription start sites (for *hrg* relevant for mRNA polyadenylation and Blastoderm-specific gene 25D or *Bsg25D*) is difficult to visualize mechanistically [39], it may involve a mechanism similar to how the human immunodeficiency virus protein Tat binds to TAR sequence in nascent mRNAs and activates transcription [40]. As an RNA-binding protein, dmPTB could regulate splicing/processing of *CG11309* (function unknown), *CG3635* (function unknown), *CanA1* (possibly involved in calmodulin activation of calcineurin), and *LpR2* (low-density lipoprotein receptor activity with potential roles in calcium ion binding and neuron projection morphogenesis) either directly or indirectly by competing for the binding of another regulatory protein, leading to alterations in the coding or non-coding regions of these transcripts. Some of these mechanisms may be similar to how the mammalian PTB regulates aspects of mRNA processing. We note that identification of downstream targets by searching for potential binding sites alone is challenging because PTB binds short, degenerate sequences UCUU and UUCU [3]–[7] that are found frequently throughout the genome. In the future, a cross-linking/immunoprecipitation experiment that detects RNA-protein interactions on a genome-wide level [41], [42] needs to be combined with our high throughput sequencing dataset to identify the subset of candidates that directly bind dmPTB.

In summary, our combined results indicate that dmPTB acts as a potent upstream regulator that specifically controls several downstream target genes and processes during embryogenesis. Many genes or transcripts altered in the *heph^03429^* mutant (Table 2) have known functional connections to specific cellular and developmental processes during embryonic development, such as neurogenesis, dorso-ventral axis formation, and dorsal closure. For others, such as down-regulation of cuticular proteins and mRNA processing defects (expected from mutation of an RNA-binding protein), functional relevance is not obvious at this stage. Genes identified in this study provide entry points and new avenues for future studies on mechanisms and biological functions as to how an RNA-binding protein controls gene expression and embryonic development.

## Materials and Methods

### Fly strains and culture

The *yellow white (yw)* stock was obtained from the Bloomington Stock Center. FRT82B *heph* ^03429^/TM6B placz was obtained from Anne Ephrussi [15]. The procedures followed for generating maternal and zygotic *heph* ^03429^ nulls are described in Besse et al., 2009. Flies were raised on standard cornmeal food at 17–25°C [43].

### RNA-Seq for gene and isoform differential expression analysis

Poly(A)^+^ RNA from 1 microgram total RNA was obtained from the *heph^03429^* mutant and wild-type control (*yw*) embryos, and mRNA-seq sequencing libraries (standard Illumina TruSeq) were prepared according to manufacturer's instructions (Illumina, Inc., San Diego CA, USA). The *heph^03429^* mutant and wild-type control libraries were sequenced on an Illumina HiSeq 2000 sequencer (singleton 100 basepair reads). TopHat 2.0.9 was used to map reads to the Flybase Drosophila melanogaster r5.44 (www.flybase.org) genome annotation (GTF and Fasta files), with the –no-novel-juncs and –microexon-search parameters (Table 1) [44]. Traces were generated using the UCSC genome browser/tools. Cufflinks version 2.1.1 (cuffdiff with the Illumina iGenomes DBGP5.25 annotation) was used to determine differential gene expression [45]. The MISO v0.4.6 [46] software package (with an isoform fraction difference threshold of 0.2 and a minimum Bayes Factor of 10) and manual inspection were used to identify differential isoform usage. Using the Drosophila annotation provided for MISO (9/19/2011), we analyzed alternative 5′ and 3′ splice sites, skipped exons, retained introns, and mutually exclusive exons (alternative first and last exons were not provided in this annotation).

### Gene Ontology analysis

The Database for Annotation, Visualization and Integrated Discovery (DAVID) v6.7 was used for gene ontology analysis online (using a differential expression threshold of four-fold). The pie charts (in Figure 2) represent the genes in only the most overrepresented ontology categories.

### *In situ* hybridization

The *in situ* protein and RNA labeling procedures followed have been described in [47] and [34]. RNA signals were detected using an Alkaline Phosphatase detection system. Protein signals were detected using a Horse Radish Peroxidase system. Embryos (0–24 hour) were collected and stage-specific embryos were sorted based on anatomy after the hybridization or antibody procedure.

### Reverse-transcription-polymerase chain reaction

Primer pairs used for RT-PCR are provided in Figure S1.

## Supporting Information

Figure S1 **The site of** ***P*** **element insertion and primer sequences are provided.**
(PDF)
