# Supplementary material for: Drosophila Polypyrimidine Tract-Binding Protein (DmPTB) Regulates Dorso-Ventral Patterning Genes in Embryos
Source: PLoS One. 2014 Jul 11;9(7):e98585. doi: 10.1371/journal.pone.0098585 (PMC4094481; doi:10.1371/journal.pone.0098585)
Supplement: Figure S1 — The site of P element insertion and primer sequences are provided. (PDF) [file pone.0098585.s001.pdf]

Supplementary Online Material:

```
                                    Genomic Sequence                                 P element insertion sequence (X06779 position 3907-3986 reverse complement)
...CCCGATTTTGACCCTCTTGCTCCTCTTTCAGTTGTATTTCCCCAGCCGTGGACACAAGTGAAACAAACATGCCATGTAC|CATGATGAAATAACATAAGGTGGTCCCGTCGGCAAGAGACATCCACTTAACGTATGCTTGCAATAAGTGCGAGTGAAAGG...
RNA Seq reads that cross over into the P element:
   CCCGATTTTGACCCTCTTGCTCCTCTTTTAGTTGTATTTCCCCAGCCGTGGACACAAGTGAAACAAACATGCCATGTAC|CATGATGAAATAACATAAGGT
       ATTTTGACACTCTTGCTCCTCTTTCAGTTGTATTTCCCCAGCCGTGGACACAAGTGAAACAAACATGCCATGTAC|CATGATGAAATAACATAAGGTGGTC
                  CTTGCTCCTCTTTCAGTTGTATTTCCCCAGCCGTGGACACAAGTGAAACAAACATGCCATGTAC|CATGATGAAATAACATAAGGTGGTCCCGTCGGCAAG
                  CTTGCTCCTCTTTCAGTTGTATTTCCCCAGCCGTGGACACAAGTGAAACAAACATGCCATGTAC|CATGATGAAATAACATAAGGTGGTCCCGTCGGCAAG
                            TTTCAGTTGTATTTCCCCAGCCGTGGACACAAGTGAAACAAACATGCCATGTAC|CATGATGAAATAACATAAGGTGGTCCCGTCGGCAAGAGACATCCAC
                             TTCAGTTGTATTTCCCCAGCCGTGGACACAAGTGAAACAAACATGCCATGTAC|CATGATGAAATAACATAAGGTGGTCCCGTCGGCAAGAGACATCCACT
                                            CCAGCCGTGGACACAAGTGAAACAAACATGCCATGTAC|CATGATGAAATAACATAAGGTGGTCCCGTCGGCAAGAGACATCCAATTAACGTATGCTTGCA
                                                       CACAAGTGAAACAAACATGCCATGTAC|CATGATGAAATAACATAAGGTGGTCCCGTCGGCAAGAGACATCCACTTAACGTATGCTTGCAATAAGTGCGAG
                                                            GTGAAACAAACATGCCATGTAC|CATGATGAAATAACATAAGGTGGTCCCGTCGGCAAGAGACATCCACTTAACGTATGCTTGCAATAAGTGCGAGTGAAA
                                                              GAAACAAACATGCCATGTAC|CATGATGAAATAACATAAGGTGGTCCCGTCGGCAAGAGACATCCACTTAACGTATGCTTGCAATAAGTGCGAGTGAAAGG
```

RNA Seq reads from the *heph*^{03429} mutant that cross over into the P element. The genomic reference sequence shown on the upper left is within the *heph* gene on chromosome 3R from 27756335 to 27756257 (reverse complement). The P element insertion sequence on the upper right was taken from a section of the pPI25.1 transformation vector (Genbank accession #X06779). Shown below the reference sequences are 100-mer reads that span from the *heph* intronic sequence into the P element sequence. No wild-type (yw) control RNA Seq reads were found with the P element insertion sequence (based upon checking 20 nt sections of the P element insertion sequence). There did not appear to be any other P element insertion site in the *heph*^{03429} sample based on RNA Seq reads. No reads were found that spanned the other end of the P element insertion into the *heph* gene.

Supplementary Table 1. Primers used for *heph*^{03429} validation of differential expression

Gene (isoform)	Forward primer	Reverse primer
Z600	GCAAATCAGCAACGTCAAGC	TTTGCGAGCCTTGATGAAAT
CanA1	TGCTAGTCAACATCCTGAACATTTGCTC	CCGAATCTTATTCCGAATTATCTCTTTG
LpR2	GCCACTGTTCTCATCTTTGCCTGC	CTATTTCGACGTAATCATTTTCTG
CG11309	GCTCGATGCTTTCACCTGTTTGTTGGGC	GTTAAGTCGCCTGCATGCCGATAACGCT
Hrg, P1	GATACACTCTCCTGCCAAAAGTTC	TATTTAAGTTTGCTCTCCAAGAAG
Hrg, P2	TGTCAATTAACTGTTTTGCTACAC	TATTTAAGTTTGCTCTCCAAGAAG
Bsg25D, P1	AAGTCCCTGCTGAAGCTCTGCTCAC	GCAACTGGTCCTCGTCCGTATTGTCCG
Bsg25D, P2	AGTTAACAGATCCGATAATTGAGAAGCTAGC	GCAACTGGTCCTCGTCCGTATTGTCCG
zerknüllt	CCAAAGTCAGGTCCCCACGA	GGAGGTTACGCACGGGTTCA
twisted gastrulation	GCCAGTAGCTATCGATGGTTCCAC	ATCGCTCCAGATCCTGTTCAGCCT
Screw	CCACGCCATCGTCCAAACCCTGAT	ATCTAATGACATCCGCACTCCTTG
mummy	ATTTGTTACCTATTGTGTCCGTTG	AATATTCATATCATTACATGTTCT
BobA	GATGACGAGAACACAAGACTGACT	TACTAACTTAGACTACATTAAAGC
Rumi	ATGAAGGACATCAAGTGCTATTGG	CATGGAATGCGGACAGAGAGATTC
m4	ACGAGCGTCTCTCCCAGTCCTGCG	TGGCGTTGGAGGTGCTGCAGTAGA
Cpr49Ad	GCTCCTATTCCTACAACTATGAGA	GTGTTTTGTATAGACTACATTGGC
Acp1	AACTTTAGACCCCTCAACACGTTA	TTCTTTATTGGCATTATGTAGATG
Zerknüllt	CATCGGTCTGCCTCCCAACT	GGAGGTTACGCACGGGTTCA
